# Lamotrigine monotherapy for newly diagnosed typical absence seizures in children^☆^

**DOI:** 10.1016/j.eplepsyres.2008.07.016

**Published:** 2008-12

**Authors:** Gregory L. Holmes, L. Matthew Frank, Raj D. Sheth, Bryan Philbrook, John D. Wooten, Alain Vuong, Susan Kerls, Anne E. Hammer, John Messenheimer

**Affiliations:** a Neuroscience Center at Dartmouth, Dartmouth Medical School, Lebanon, NH, United States; b Eastern Virginia Medical School, Norfolk, VA, United States; c Department of Neurology, University of Wisconsin at Madison, Madison, WI, United States; d Child Neurology Associates, P.C., Atlanta, GA, United States; e Raleigh Neurology Associates, Raleigh, NC, United States; f GlaxoSmithKline, Research Triangle Park, NC, United States

**Keywords:** Clinical trial, Absence seizures, Antiepileptic drugs

## Abstract

**Purpose:**

To evaluate the efficacy, tolerability, and effects on behavior and psychosocial functioning of lamotrigine monotherapy in children with newly diagnosed typical absence seizures.

**Patients and methods:**

Children meeting enrollment criteria (*n* = 54) received a confirmatory 24-h ambulatory electroencephalogram (EEG) and then entered a Escalation Phase of up to 20-weeks during which lamotrigine was titrated until seizures were controlled or maximum dose (10.2 mg/kg) was reached. Seizure freedom was assessed by diary review and clinic hyperventilation (clinic HV) and then confirmed by EEG with hyperventilation (HV/EEG). Patients who maintained seizure freedom for two consecutive weekly visits were entered into the Maintenance Phase (*n* = 30). Diary, clinic HV, and HV/EEG data were supplemented with 24-h ambulatory EEG at baseline and the ends of the Escalation and Maintenance Phases. Health outcome assessments were completed at screening and at the end of the Maintenance Phase.

**Results:**

By the end of the Escalation Phase, seizure-free rates (responders) were 59% by seizure diary (*n* = 51), 56% by HV/EEG (*n* = 54) (primary endpoint), and 49% by 24-h ambulatory EEG (*n* = 49). During the Maintenance Phase, 89% (week 24) and 86% (week 32) remained seizure free by diary (*n* = 28), 78% by clinic HV (*n* = 27), and 81% by 24-h ambulatory EEG (*n* = 26). Seizure freedom was first observed beginning at the fifth week of the Escalation Phase. The most frequent adverse events were headache and cough. Health outcome scores were either improved or unchanged at the end of the Maintenance Phase.

**Conclusions:**

Lamotrigine monotherapy results in complete seizure freedom in a substantial number of children with typical absence seizures. Lamotrigine was well tolerated in this study.

## Introduction

Typical absence seizures, which account for approximately 10% of seizures in children, are characterized by brief (<30 s), sudden, and unpredictable episodes of impairment of consciousness (absence) associated with generalized spike/polyspike-and-slow-wave discharges on electroencephalogram (EEG) and often accompanied by motor automatisms (Panayiotopoulos, 2001; Posner, 2005). Patients with absence epilepsy routinely experience multiple seizures per day, sometimes numbering in the hundreds (Pearl and Holmes, 2001). Typical absence seizures occur spontaneously or can be provoked by various triggers such as photic stimuli or hyperventilation. Usually, onset of typical absence seizures occurs in childhood or adolescence, and seizures remit with age in many patients (Adams and Lueders, 1981; Wirrell et al., 1996a,b; Pearl and Holmes, 2001; Wirrell, 2003; Grosso et al., 2005; Tovia et al., 2006). Given that frequent, often subtle seizures can cause difficulty with accurate seizure counts and assessment, hyperventilation during EEG monitoring is an important tool in the diagnosis of typical absence seizures (Browne et al., 1983; Wirrell et al., 1997).

Effective control of typical absence seizures is important given their potential impact on children's safety as well as behavior, cognition, and psychosocial function during a critical period of development. Typical absence seizures may affect children's safety by increasing risk of accidental injury. In a study of children and adolescents <18 years old diagnosed with either typical absence epilepsy or juvenile rheumatoid arthritis (a control group defined by having a non-neurological chronic disease), the risk of accidental injury, particularly mild head injuries and injuries from bicycle accidents or car accidents, was significantly higher in patients with typical absence epilepsy than patients with juvenile rheumatoid arthritis (Posner, 2005; Posner et al., 2005a).

A 2005 Cochrane review of clinical trials of ethosuximide, valproate, and lamotrigine in typical absence seizures in children identified only five small, randomized, prospective studies (Posner et al., 2005b). Thus, while lamotrigine, valproate, and ethosuximide are each regarded as effective for typical absence seizures, little information from clinical trials is available to inform their use for typical absence seizures in clinical practice.

The study reported herein was designed as an evaluation of the efficacy of lamotrigine monotherapy for the treatment of typical absence seizures in children. This study design was open-label with a step-wise escalation of lamotrigine, using the Food and Drug Administration (FDA) guideline for rate of escalation. Our goals were to learn how many children would become seizure free on lamotrigine monotherapy, the dose at which lamotrigine was efficacious, and whether there were EEG or behavior predictors of response to lamotrigine.

## Methods

### Patients

Male or female patients <13 years old who were newly diagnosed with absence epilepsy and had not previously been treated with antiepileptic drugs were eligible for the study. Presence of typical absence seizures was suspected on the basis of hyperventilation in the clinic and confirmed by observance of clinical and electroencephalographic features (i.e., 2.5–4.5 Hz generalized spike-and-wave activity or multiple spike-and-wave activity lasting ≥3 s while awake) of typical absence seizures on a 1-h EEG with hyperventilation with two 5-min trials (HV/EEG). All the children met the requirements of childhood absence epilepsy (Pearl and Holmes, 2008). The definition of a seizure as an electroencephalographic discharge of 3 s or longer was arbitrary. However, most children with electrographic discharges lasting this long will have altered responsiveness (Porter et al., 1973; Browne et al., 1974; Pearl and Holmes, 2001). All screening HV/EEGs were also read centrally by an electroencephalographer (GLH) to confirm the diagnosis.

Exclusion criteria included seizures resulting from an identifiable intracerebral lesion; the presence of partial or generalized tonic–clonic seizures, a progressive neurological disorder; clinically significant chronic hepatic, renal, or cardiac conditions; a psychiatric disorder requiring medication; a history of a severe psychiatric disorder requiring hospitalization; current use of psychoactive drugs to treat attention-deficit hyperactivity disorder; and pregnancy, breastfeeding, or inability to confirm sexual abstinence.

### Procedures and assessments

The protocol for this open-label study was approved by institutional review boards for the 19 investigational sites in the United States. All children and/or their parents or caregivers provided written informed consent.

The study consisted of a 1-week Screening Phase, a 24-h Baseline Phase, a 20-week Escalation Phase, and a 12-week Maintenance Phase. During the Screening and Baseline Phases, patients were evaluated to determine whether they met enrollment criteria. During the Escalation Phase, clinic visits occurred on the second and fourth weeks of treatment and weekly thereafter. Patients who met enrollment criteria received The study consisted of a Screening Phase of up to 1 week, a 24-h Baseline Phase, an Escalation Phase of up to 20 weeks, and a 12-week Maintenance Phase. During the Screening and Baseline Phases, patients were evaluated to determine whether they met enrollment criteria. Ambulatory EEG recording was carried out during the 24-h Baseline Phase. During the Escalation Phase, clinic visits occurred on the second and fourth weeks of treatment and weekly thereafter. Patients who met enrollment criteria received lamotrigine monotherapy titrated according to the schedule in Table 1 (starting dose: 0.3 mg/(kg day)) until the patients became seizure free or the 20-week Escalation Phase was complete (10.2 mg/(kg day) maximum dose). At each clinic visit, seizure status was assessed by hyperventilation testing (two 5-min trials) for clinical signs (clinic HV) and, if the results were either equivocal or negative, an EEG with hyperventilation was performed. Freedom from seizures was defined as no absence seizure discharges during the EEG with hyperventilation on two consecutive weeks. Between visits, a single dose increment of 0.6 mg/kg occurred. A 24-h ambulatory EEG followed the second seizure-free HV/EEG.

Patients who did not become seizure free during the Escalation Phase were discontinued from the study. At the end of the Escalation Phase, all patients underwent a 24-h ambulatory EEG recording prior to entering the Maintenance Phase (patients whose seizures were controlled) or discontinuing from the study (patients whose seizures were not controlled).

Only patients who met the criterion for seizure freedom entered the Maintenance Phase, during which they remained for an additional 12 weeks at the effective dose established during the Escalation Phase unless dose adjustment (maximum: 15.0 mg/(kg day); minimum: 0.3 mg/(kg day)) was required to optimize efficacy or tolerability. Clinic visits occurred every 4 weeks during the Maintenance Phase. Clinic HV was performed at visits in the Maintenance Phase.

At the end of the Maintenance Phase, a third 24-h ambulatory EEG was performed. In addition, investigators rated the patient's status relative to status at Screening on seven indices (Seizure Frequency, Seizure Duration, Seizure Intensity, Adverse Experiences, Social Functioning, Intellectual Functioning, and Motor Functioning), each scored on a 7-point scale: marked deterioration, moderate deterioration, mild deterioration, no change, mild improvement, moderate improvement, marked improvement. Parents/caregivers also used this 7-point scale to assess the child's overall status at the end of the Maintenance Phase.

Health outcomes assessments completed at Screening and at the end of the Maintenance Phase included the Child Behavior Checklist for Ages 6–18 (CBCL) (Shaffer et al., 1983; Harter, 1985; Bird et al., 1987; Granleese and Joseph, 1994), the Children's Global Assessment (C-GAS)(Achenbach, 1991), and the Self-Perception Profile for Children (SPPC) (Bird et al., 1987). The CBCL is a numeric scale (1–100) used by mental health clinicians and doctors to rate the general functioning of children under the age of 18. The checklist captures information on competencies and behavioral/emotional problems in the domains of Anxious/Depressed, Withdrawn/Depressed, Somatic Complaints, Social Problems, Thought Problems, Attention Problems, Rule-Breaking Behavior, and Aggressive Behavior (Harter, 1999). Parents/caregivers were asked to rate their children's status over the past 6 months on each domain on a 3-point scale: 0 = not true; 1 = somewhat or sometimes true; 2 = very true or often true. The CBCL was summarized as a total score and as scores for each of the subscales. Total scores range from 0 to 224. Higher scores reflect lower competencies and greater emotional/social problems. The C-GAS is a single-item assessment of overall general functioning for children ages 4 and older (Bird et al., 1987). Parents/caregivers were asked to identify the category that best represents their children's current functioning and then to specify a value on a 100-point scale that best corresponded to that level. The C-GAS yields a single score ranging from 0 to 100 where higher scores reflect better functioning. The SPPC is an interviewer-administered questionnaire to assess perception of competence and adequacy across the domains of athletic competence, behavioral conduct, global self-worth, physical appearance, scholastic competence, and social acceptance (Harter, 1999). The SPPC was summarized as one score for each domain for a total of six scores, each ranging from 1 to 4. Higher scores reflect better competence or self-perception.

Throughout the study, all patients and their parents/caregivers kept seizure diaries for recording presence or absence of seizures each day. Adverse events, defined as any untoward medical occurrences reported by patients or their parents/caregivers or noted by investigators, were also recorded throughout the study. Adverse events were reported regardless of their cause. For each adverse event, investigators recorded whether or not they considered it to be caused by study medication and whether or not it was serious. A serious adverse event was defined as any untoward experience, regardless of its suspected cause, that was fatal, life-threatening, or permanently disabling; or that required inpatient hospitalization.

Serum samples to measure lamotrigine concentrations were collected at the time of confirmation of seizure freedom or at the time of premature withdrawal from the study.

For patients discontinuing the study for any reason, lamotrigine dose was reduced by approximately 50% per week over at least 2 weeks unless safety concerns dictated a more rapid withdrawal of study medication. The use of psychoactive drugs to treat hyperactivity disorder or attention-deficit disorder or antiepileptic drugs other than study medication was prohibited throughout the study.

### Data analysis

Efficacy data were analysed for subjects contributing data in each phase. All available data up to the time of study discontinuation were included in analyses of efficacy data for patients who withdrew from the study prematurely. Missing data were not interpolated.

The primary efficacy endpoint was the proportion of patients with no typical absence seizures for two consecutive weeks as confirmed by HV/EEG during the Escalation Phase. An absence seizure was defined as a spike-and-wave or polyspike-and-wave discharge lasting ≥3 s during the awake state. This arbitrary definition was based on studies showing that generalized discharges of this duration are associated with impaired function (Porter et al., 1973; Browne et al., 1974; Penry et al., 1975). A patient was considered seizure-free at a given week if confirmed seizure-free the following week.

Other efficacy endpoints, calculated separately for the Escalation and Maintenance Phases, included the percentage of patients seizure free on 24-h ambulatory EEG; the mean number of seizures per 24 h by 24-h ambulatory EEG; the percentages of patients with ≥25%, ≥50%, ≥75%, and ≥100% reductions versus Baseline in seizure frequency and seizure duration by 24-h ambulatory EEG; the percentages of patients with ≥25%, ≥50%, ≥75%, and ≥100% reductions versus Baseline in clinical signs of absence seizures on hyperventilation; the mean percent change versus Baseline in days per week with seizures by seizure diary; and the percentage of patients seizure free according to diary data. For the seizure diary data, Baseline values for days per week with seizures were based on the number of days with seizures during the 4 weeks preceding study entry. Other endpoints included the percentages of patients scored as moderately or markedly improved on the investigator and parent/caregiver ratings of patients’ status; mean changes from Baseline to the end of the study (defined as the last visit on study medication) on health outcomes measures; the percentage of patients with adverse events; and serum lamotrigine concentration at the end of the Escalation Phase in the sample as a whole, seizure-free patients (responders), and patients not seizure free (non-responders).

For the percentages of patients seizure free by HV/EEG (primary endpoint) and by 24-h ambulatory EEG, exact binomial two-tailed tests for a single proportion were used to compare seizure-free rates in this lamotrigine-treated sample with a null-hypothesis value of 20%, which represented a historical seizure-free rate in a placebo group. The historical placebo response rate of 20% was based on the observed seizure-free rate in the placebo group during the 4-week Maintenance Phase of the previously reported controlled clinical trial of lamotrigine for typical absence seizures in children (Frank et al., 1999). This seizure-free rate may overestimate the true placebo rate because it was obtained while patients who had been successfully treated with lamotrigine were slowly tapered off lamotrigine to placebo. Based on published findings on the natural history of absence epilepsy, a seizure-free rate of 20% within the time frame of the current study would constitute a very high remission rate for untreated patients following recent diagnosis (Ferrie et al., 1995; Buoni et al., 1999; Coppola et al., 2004a,b). Paired two-tailed *t*-tests were used to compare Escalation-Phase values and Maintenance-Phase values with Baseline values for mean percent change in weekly seizure frequency by seizure diary and mean changes in scores on the health outcomes measures. All other data were summarized with descriptive statistics only.

According to power calculations using an estimated 50% seizure-free rate with lamotrigine and a significance level of 0.05, a total of 35 evaluable patients provided at least 90% power to detect a significant difference between lamotrigine and a historical placebo group with a seizure-free rate of 20% for the primary endpoint. Because a 30% dropout rate was assumed, planned enrollment was approximately 50 patients in order to obtain 35 evaluable patients.

## Results

### Patients

The number of patients enrolled in the study was 54, all of whom took at least one dose of study medication and were therefore included in the Intent-to-Treat Population. Two thirds of the sample was female, and the mean age of the patients was 7.3 years (range 3–13) (Table 2).

Of the 54 patients enrolled in the study, 28 (52%) completed the study and 26 (48%) prematurely withdrew. The primary reason for premature withdrawal was lack of efficacy (Table 2). All withdrawals for lack of efficacy (i.e., 21 of the total 26 premature withdrawals in the study) occurred at the end of the Escalation Phase because patients met the exit criterion of not being seizure free at the end of that phase.

### Seizure status

#### HV/EEG

At the end of the Escalation Phase, thirty patients were seizure free by HV/EEG (primary endpoint) (30/54, 56%, *p* < 0.0001 versus a historical 20% incidence of seizure freedom with placebo). Seizure freedom occurred in some patients beginning on the fifth week of the Escalation Phase.

#### 24-h Ambulatory EEG

Of the 49 patients with evaluable ambulatory EEG data at the end of the Escalation Phase, 24 (49%) were seizure free (*p* < 0.0001 versus historical 20% placebo rate). Of the 26 patients with evaluable ambulatory EEG data at the end of the Maintenance Phase, which consisted only of patients who achieved seizure freedom during the Escalation Phase, 21 (81%) were seizure free (*p* ≤ 0.001 versus historical 20% placebo rate). Of the 54 patients entered into the study, 21 (39%) reached the Maintenance Phase and remained seizure free. The mean ± S.D. number of absence seizures per 24 h was 25.6 ± 44.5 at the end of the Escalation Phase and 6.3 ± 16.8 at the end of the Maintenance Phase compared with 60.4 ± 49.9 at Baseline. During both the Escalation Phase and the Maintenance Phase, the majority of patients had at least a 75% reduction in seizure frequency and seizure duration on 24-h ambulatory EEG. Table 3 lists the percentage of patients with reduction in spike-wave discharges by 25%, 50%, 75%, and 100% in patients during the escalation and Maintenance Phases.

#### Clinic HV

At Baseline, additional clinical signs of absence seizures on hyperventilation included staring (80% of patients), impairment of consciousness (69%), eye blinking (50%), eye rolling (46%), chewing movements (20%), hand movements (15%), and other automatisms (7%). During the Escalation Phase, 29 of 53 patients had at least a 25% reduction in clinical signs of absence seizures (Table 3). During the Maintenance Phase, which consisted only of patients who achieved seizure freedom during the Escalation Phase, all 27 patients had at least a 25% reduction in clinical signs of absence seizures, and 6 of 27 patients had a 100% reduction (Table 3).

#### Seizure diary data

The mean ± S.D. percent change versus Baseline in days per week with seizures by seizure diary was −25.2% ± 171.39 (*p* = 0.2979 versus Baseline) during the Escalation Phase and −95.5% ± 13.85 during the Maintenance Phase (*p* < 0.0001 versus Baseline). By seizure diary, approximately half 23 of 51 patients were seizure free by week 14 of treatment, and 25 of 28 (week 24) and 24 of 28 (week 32) patients were seizure free during the Maintenance Phase.

### Characteristics of responders and non-responders to lamotrigine

To determine if there were factors that predicted response to lamotrigine, we evaluated clinical and electroencephalographic features in lamotrigine responders (no seizures) and non-responders. There were no differences in mean age (responders 7.2 ± 2.6 years; non-responders 7.5 ± 2.9 years), baseline number of days per week with seizures (responders 6.0 ± 2.1; non-responders 6.1 ± 2.3), baseline seizure number on the pre-drug 24 h EEG (responders 57.8 ± 49.3 seizures; non-responders 63.7 ± 51.4 seizures), baseline total seizure duration on the pre-drug 24 h EEG (responders 532.5 ± 436 s; non-responders 662.8 ± 467.8 s) and baseline total duration of absence seizures (responders 617.45 ± 426.75 s; non-responders 594.00 ± 454.59 s, *p* > 0.05). There were also no differences in responder rate between young children (3–7 years; 14 of 28 patients responsed) and older children (8–13 years; 16 of 26 patients responsed).

### Investigator and parent/caregiver ratings of patient's status

On the investigator rating of clinical status, the percentage of patients scored as showing moderate or marked improvement from the beginning of the study to the end of the Maintenance Phase was 97% for Overall Status, 100% for Seizure Frequency, 97% for Seizure Duration, 96% for Seizure Intensity, 28% for Adverse Experiences, 37% for Social Functioning, 41% for Intellectual Functioning, and 27% for Motor Functioning (Table 4). On the parent/caregiver assessment of overall status, 100% of patients were rated as showing moderate or marked improvement from the beginning of the study to the end of the Maintenance Phase.

### Health outcomes

The mean ± S.D. change from Baseline to the end of the study in CBCL total score reflected a significant improvement in behavior (−10.8 ± 17.3; *p* = 0.0009). Mean ± S.D. changes from Baseline also reflected significant improvement in the domains of Anxious/Depressed (−1.2 ± 2.5, *p* = 0.010), Aggressive Behavior (−1.7 ± 3.1, *p* = 0.003), Social Problems (−1.7 ± 2.3, *p* < 0.001), Thought Problems (−1.6 ± 2.9, *p* = 0.003), and Attention Problems (−2.2 ± 5.0, *p* = 0.017). Mean changes from Baseline did not reflect statistically significant improvement in the domains of Withdrawn/Depressed, Somatic Complaints, or Rule-Breaking Behavior.

Mean ± S.D. C-GAS scores did not significantly differ between Baseline (86.7 ± 9.5, reflecting good functioning) and the end of the study (89.0 ± 8.0). Likewise, mean changes from Baseline to the end of the study in SPPC subscale scores were not statistically significant except for social acceptance (2.1, *p* = 0.046).

### Adverse events

The most common adverse events reported during the study were headache and cough (Table 5). Rash was reported in six patients (11%) subjects, urticaria in one patient (2%), and pruritus in two patients (4%). None of the incidents of rash were considered to be drug related; the urticaria and pruritus were considered possibly to be drug related. None of these events was serious or resulted in premature withdrawal from the study.

Three patients had adverse events that led to premature withdrawal from the study: increased seizure activity in one patient, tremor in one patient, and vomiting and dizziness in one patient. In the investigators’ judgment, all of these adverse events were possibly caused by lamotrigine. One of the adverse events, increased absence seizure activity in a 4-year-old male patient, was considered to be serious. This event was observed beginning 16 weeks after initiation of lamotrigine and 2 days after dose adjustment to 8.74 mg/(kg day). The patient was hospitalized for evaluation while lamotrigine was discontinued and ethosuximide was initiated. The event resolved by 3 days after its onset.

### Lamotrigine serum concentrations

Among the 29 patients with available data on lamotrigine serum concentrations, mean ± S.D. (range) trough serum concentration at the end of the Escalation Phase was 8.4 ± 4.3 μg/mL (2.9–20.8). At the end of the Escalation Phase, 19 of the 29 patients with lamotrigine serum-concentration data were seizure free, and the remaining 10 patients were not seizure free. Mean ± S.D. (range) trough serum concentration at the end of the Escalation Phase was 6.3 ± 2.2 μg/mL (2.9–10.4) among the 19 seizure-free patients and 12.5 ± 4.5 μg/mL (3.5–20.8) among the 10 patients who were not seizure free.

## Discussion

This EEG-based study supports a role for lamotrigine in the treatment of typical absence seizures in children and adolescents. Complete seizure control by HV/EEG occurred in some lamotrigine-treated patients beginning on the fifth week of the Escalation Phase. By the end of the Escalation Phase, seizure-free rates were 59% by seizure diary, 56% by HV/EEG, and 49% by 24-h ambulatory EEG. Seizure control was largely maintained during the 12-week Maintenance Phase, during which the percentage of patients who remained seizure free was 89% (week 24) and 86% (week 32) by seizure diary, 78% by clinic HV, and 81% by 24-h ambulatory EEG. Lamotrigine was also associated with improvement in patients’ clinical status and behavioral functioning as rated by both investigators and parents/caregivers and as assessed on the CBCL.

Mean trough serum concentration at the effective dose of lamotrigine among patients seizure free was 6.3 μg/mL. The finding that lamotrigine serum concentrations were approximately two times higher among patients who were not seizure free as those who were seizure free (12.5 μg/mL versus 6.3 μg/mL) suggests that inadequate dose was not a reason for lack of response to lamotrigine in those patients who did not become seizure free by the end of the Escalation Phase.

This study extends the results of previous open-label research supporting the efficacy of lamotrigine for absence seizures (Kluger et al., 2001; Duchowny et al., 2002; Brodbeck et al., 2006), as well as findings of a placebo-controlled trial of lamotrigine monotherapy in newly diagnosed typical absence seizures in children (Frank et al., 1999). This study adds to the current literature on lamotrigine in absence seizures by demonstrating that efficacy was seen by the fifth week off therapy. Because there is evidence that slow titrations of lamotrigine reduce the risk of serious rash with lamotrigine (Messenheimer, 1998; Hirsch et al., 2006), in this study we elected to mimic clinical practice and used an open-label approach where the dosage escalation was identical to that approved for package labeling by the FDA. Because of this slow titration there was concern that efficacy would not be seen until a target dose of 5–10 mg/kg was reached. However, some patients started responding at doses as low as 1.2 mg/kg.

Effective control of typical absence seizures is important given their potential impact on children's safety as well as behavior, cognition, and psychosocial function during a critical period of development. Typical absence seizures may affect children's safety by increasing risk of accidental injury. In a study of children and adolescents <18 years old diagnosed with either typical absence epilepsy or juvenile rheumatoid arthritis (a control group defined by having a non-neurological chronic disease), the risk of accidental injury, particularly mild head injuries and injuries from bicycle accidents or car accidents, was significantly higher in patients with typical absence epilepsy than patients with juvenile rheumatoid arthritis (Posner, 2005; Posner et al., 2005a). Data from these cohorts also suggest that absence seizures can cause persistent deficits in behavioral and psychosocial functioning. At a follow-up interview conducted when patients were 23 years old on average, those with typical absence epilepsy had poorer functioning in the academic-personal and behavioral domains than patients with juvenile rheumatoid arthritis (Frank et al., 1999; Posner, 2005, 2006; Posner et al., 2005a). Outcomes were poorest among those with ongoing absence seizures.

For these reasons, we set very high criteria for entrance into the Maintenance Phase, total seizure freedom by HV EEG maintained over 2 weeks. Because absence seizures can be subtle and missed by parents and caregivers, to assure that we were achieving total seizure control we required 24 h EEGs at baseline and again following the end of the Escalation Phases in children. We anticipated that the 24 h EEG would detect seizure events not observed by parents or detected in a 1 h EEG. Indeed, we found that the patient diaries and EEG with hyperventilation over-estimated the number of children who were seizure free, when compared to the 24 h EEG. While total seizure control was seen in less than half of the children, based on the 24 h EEG, it should be noted that the mean number of absence seizures per 24 h decreased from 60.4 ± 49.9 at baseline to 25.6 ± 44.5 at the end of titration. While reducing seizure frequency by greater than 50% is certainly beneficial, the goal is to obtain total seizure control, hence the termination of the study in the lamotrigine non-responders. Coppola et al. (2004a,b) also required 100% seizure control in their cohort of children with typical absence seizures who received either valproate or lamotrigine monotheraphy in open-label studies. As in this study Coppola et al. (2004a,b) used 24 h EEG records to confirm seizure freedom although it was not clear what EEG criteria was used to classified the electrical discharges as seizures.

Limitations of this study should be considered in interpreting the data. The study was of open-label design and did not include a placebo control group. The impact of these limitations on the primary endpoint is arguably negligible given that absence seizures on EEG are reliably identified and should be subject to little if any misinterpretation. To compensate for the lack of a placebo control group, the seizure-free rate in the lamotrigine-treated patients in the current sample was compared with a historical placebo response rate of 20%, which was based on the observed seizure-free rate in the placebo group in the controlled clinical trial of lamotrigine for typical absence seizures. This seizure-free rate on placebo was observed in patients who converted from open-label lamotrigine to double-blind treatment with placebo after achieving seizure-freedom on lamotrigine monotherapy. The observed rate at which these patients maintained their seizure control over 4 weeks of placebo treatment and concomitant taper of lamotrigine probably overestimates the true placebo response that would have been observed with placebo without concomitant lamotrigine. The seizure-free rate with lamotrigine in the current study was significantly higher than the 20% historical placebo response, which would constitute a high remission rate for newly diagnosed patients.

Lamotrigine was generally well tolerated in this study. The pattern and incidence of adverse events were consistent with those in previous studies of children and adults. No cases of serious rash were reported. In the clinical development program for lamotrigine, a 10% rate of non-serious rash reported in most clinical trials, in line the findings from this study. Investigators were instructed to deem a rash drug related unless a specific cause could be identified. In this study, none of the investigators considered rash drug related. However, even with the slow titration rate used here it is important for the clinician to be aware of serious dermatological responses to lamotrigine, including Stevens Johnson syndrome and toxic epidermal necrolysis (Guberman et al., 1999; Hirsch et al., 2006).

Lamotrigine therapy was associated with improvements in the Children's Behavior Checklist although the Children's Global Assessment and Self-Perception Profile for Children did not change from baseline. Only patients who became seizure-free at the end of dose escalation had health outcomes measured. Since seizure frequency, duration and intensity improved during the course of study it is somewhat surprising that there were no changes in the latter two tests. While these were healthy children with newly onset seizures, it is likely that these children were well generally adjusted at the time of entry into the study and that a ceiling effect occurred. However, the investigator rating of patients’ status showed significant improvements in overall status, social functioning, and motor functions while in the parents’ assessment 100% of the patients were rated as showing moderate or marked improvement from the beginning of the study to the Maintenance Phase. While these results must be interpreted cautiously in view of the lack of a placebo control group, they do support the view that lamotrigine is well tolerated and has some beneficial effects on behavior, motor function, and social skills.

In conclusion, lamotrigine monotherapy was effective and well tolerated for the treatment of typical absence seizures in newly diagnosed patients. Lamotrigine is an important therapeutic option for managing typical absence seizures. This study does not answer the question of which drug is the best in regards to efficacy and tolerability for typical absence seizures. Whether lamotrigine is more effective than other antiepileptic drugs in typical absence seizures is not clear. In the Standard and New Antiepileptic Drugs (SANADs) trial, valproate was found to be superior to lamotrigine in an unblinded, randomized trial (Marson et al., 2007). Unfortunately, there was insufficient power to allow the authors to make definite statements about the relative efficacy and effectiveness of the drugs for individual seizure types and sub-syndromes within the idiopathic generalized epilepsies. The answer to the question of the best drug for typical absence seizures will have to await a large, multicenter comparison study of lamotrigine, ethosuximide, and valproate in absence seizures currently being conducted by the National Institutes of Health.

## Figures and Tables

**Table 1:** Dose-escalation schedule for lamotrigine

Treatment week	Dose (mg/(kg day))^a^
1–2	0.3^b^
3–4	0.6
5	1.2
6	1.8
7	2.4
8	3.0
9	3.6
10	4.2
11	4.8
12	5.4
13	6.0
14	6.6
15	7.2
16	7.8
17	8.4
18	9.0
19	9.6
20	10.2

a Patients entered the Maintenance Phase upon reaching their efficacious dose as determined by confirmed seizure freedom at two consecutive visits.

b This dose was given in one dose or two divided doses.

**Table 2:** Demographics and patient disposition

	*n* = 54
Demographics	
Mean age, years (range)	7.3 (3–13)

Sex, *n* (%)	
Female	34 (63)
Male	20 (37)

Race, *n* (%)	
African American/African heritage	15 (28)
American Indian or Alaska native	4 (7)
Japanese	1 (2)
White—Arabic/North African heritage	1 (2)
White—White/Caucasian/European heritage	33 (61)

Patient disposition	
Completed the study, *n* (%)	28 (52)

Prematurely withdrew from the study, *n* (%)	26 (48)
Escalation Phase, *n*	24
Lack of efficacy^a^	21
Adverse event	2
Lost to follow-up	1
Patient's decision	0

Maintenance Phase, *n*	2
Lack of efficacy	0
Adverse event	1
Lost to follow-up	0
Patient's decision	1

a These patients met the exit criterion of not being seizure free at the end of the Escalation Phase.

**Table 3:** Percent of patients with ≥25%, ≥50%, ≥75%, and 100% reduction in seizure frequency and duration on 24-h ambulatory EEG or Clinic HV at the end of escalation and maintenance, which consisted only of patients who achieved seizure freedom during the Escalation Phase

	Seizure frequency on 24-h ambulatory EEG	Seizure duration on 24-h ambulatory EEG	Clinic HV
Escalation Phase	*n* = 46	*n* = 46	*n* = 53
≥25% reduction	76	76	55
≥50% reduction	72	74	26
≥75% reduction	59	61	4
100% reduction	48	48	0

Maintenance Phase	*n* = 26	*n* = 26	*n* = 27
≥25% reduction	88	92	100
≥50% reduction	81	92	93
≥75% reduction	81	81	85
100% reduction	81	81	78

**Table 4:** Investigators’ global assessment at the end of the Maintenance Phase

	Deterioration			No change	Improvement		
	Marked	Moderate	Mild		Mild	Moderate	Marked
Overall status	0 (0)	0 (0)	0 (0)	0 (0)	1 (3)	6 (21)	22 (76)
Seizure frequency	0 (0)	0 (0)	0 (0)	0 (0)	0 (0)	2 (7)	27 (93)
Seizure duration	0 (0)	0 (0)	0 (0)	0 (0)	1 (3)	1 (3)	27 (93)
Seizure intensity	0 (0)	0 (0)	0 (0)	0 (0)	1 (3)	1 (3)	27 (93)
Adverse experiences	0 (0)	0 (0)	3 (10)	18 (62)	0 (0)	0 (0)	8 (28)
Social functioning	0 (0)	0 (0)	1 (3)	14 (48)	3 (10)	1 (3)	10 (34)
Intellectual functioning	0 (0)	0 (0)	1 (3)	13 (45)	3 (10)	3 (10)	9 (31)
Motor functioning	0 (0)	0 (0)	1 (3)	17 (59)	3 (10)	1 (3)	7 (24)

Data are expressed as *n* (% patients); *n* = 29.

**Table 5:** Number (%) of patients with adverse events

	Number (%) patients, *n* = 54
Headache	20 (37)
Cough	12 (22)
Upper abdominal pain	10 (19)
Nasal congestion	10 (19)
Nasopharyngitis	8 (15)
Pyrexia	7 (13)
Rash	6 (11)
Viral gastroenteritis	5 (9)
Dizziness	5 (9)
Psychomotor hyperactivity	5 (9)
Nausea	4 (7)
Pain in extremity	4 (7)
Pharyngolaryngeal pain	3 (6)
Constipation	3 (6)
Stomach discomfort	3 (6)
Vomiting	3 (6)
Streptococcal pharyngitis	3 (6)
Sinusitis	3 (6)

Adverse events reported in ≥5% of patients are listed.
